# Neoliberal economic policies as a root cause of forced migration from Arab Spring countries: the case of Syria

**DOI:** 10.1111/disa.70025

**Published:** 2025-10-16

**Authors:** Ahmad AL Ajlan

**Affiliations:** ^1^ Faculty of Sociology Bielefeld University Germany

**Keywords:** Arab Spring countries, civil war, forced migration, International Monetary Fund (IMF), neoliberal economic policies, Syria, World Bank

## Abstract

This article illustrates the impact of neoliberal economic policies on forced migration from Arab Spring countries. It highlights how these policies, based on the recommendations of the International Monetary Fund and the World Bank, significantly contributed to the outbreak of civil wars and subsequent refugee crises. The central argument of this analysis is that the economic reform programmes were a primary driver of the forced migration wave after 2010. Using Syria as a case study, the paper shows how the implementation of these programmes led to the erosion of the social contract that had previously ensured stability in Syria. This erosion was characterised by the marginalisation of rural areas and populations, the reduction of subsidies, and the economic and political marginalisation of the country's youth. The article calls on international financial institutions to take into consideration the nature of political regimes and the dynamics of authoritarianism to prevent turmoil, civil wars, and forced migration in the future.

## 1 INTRODUCTION

Although the world faces a forced displacement crisis in which tens of millions of individuals have had to cross international boundaries worldwide, one finds few studies on the ‘root causes’ of forced migration waves. Several researchers, including Cummings et al. (2015), have pointed out that while the literature on drivers of migration, in general, is substantial, few works specifically examine the factors propelling irregular migration. Furthermore, Schmeidl (1997) noted that most studies focus exclusively on voluntary migration and thus provide little guidance for the analysis of refugee movements. Moreover, research exploring ‘refugeeism’ in greater detail often centres on single refugee streams and describes such populations once they reach the asylum country; at times, it neglects the causes. Davenport, Moore, and Poe (2003) concluded that most studies of forced migration focus on the experiences of the people as they seek new living arrangements and the challenges associated with aiding them. The literature on the aetiology of forced migrant flows is relatively small and largely idiographic.

Yet, as Cummings et al. (2015) found, an effective and durable policy response to the current ‘migration crisis’ in Europe requires a better understanding of the causes of migration more broadly. In line with this thinking, Kang (2021) confirms that, to tackle the root causes of asylum‐seeking, the European Union (EU)'s asylum policy must be extended to the area of push factors.

In the Global South, many countries have introduced what are often called economic reform policies, or structural adjustment programmes, designed by the International Monetary Fund (IMF) and the World Bank, such as Egypt (Hazell et al., 1995), Kenya (Githua, 2013), Nigeria (Isiani et al., 2021), and Pakistan (Khan, Nawaz, and Hussain, 2011). We know very little, however, about the impact of such policies/programmes on state instability in these nations.

The IMF is a powerful international financial institution. Founded in the aftermath of the Second World War, its basic purposes were to facilitate world trade and promote national prosperity. Soon after its inception in 1945, the IMF became involved with developing countries. This has grown to such an extent that most developing states have participated in its economic reform programmes. These grant governments access to loans, but this can be swiftly cut off if they fail to comply with specific policy conditions (Vreeland, 2007).

Although the IMF works closely with the World Bank and these actors exhibit many common characteristics, they have some differences. For instance, the World Bank's declared role is to reduce poverty by lending money to the governments of its poorer members to improve their economies and the standard of living of their people, but the main function of the IMF is to assist its members—both industrialised and developing countries—that find themselves confronting temporary balance of payments difficulties, by providing short‐ to medium‐term credits. Thus, the main working area of the World Bank is promoting the economic development of the planet's poorer countries through long‐term financing of development projects and programmes (Driscoll, 1995).

Macroeconomic reforms under the auspices of both the IMF and the World Bank have been implemented in the Arab region since the 1990s (Hopfinger, 1996). The Syrian Arab Republic is a case in point. It is an agricultural developing country with a semi‐arid climate and a high population growth rate (Yassin, 2011; Bayram and Gök, 2020). Situated at the eastern end of the Mediterranean Sea, it spans a total area of 185,180 square kilometres and is bordered in the north by Turkey, in the west by Lebanon, in the east by Iraq, and in the south by Israel and Jordan. After a period under French mandate (1923–46), Syria achieved independence on 17 April 1946.

The government launched an economic liberalisation programme in 1991 to pull the country out of a financial crisis (Mora and Wiktorowicz, 2003). It expanded and intensified its economic reform policies from 2000, including opening the market to foreign investment and granting licences to foreign banks to operate in Syria (Raphaeli, 2007). The implications set the scene for the uprising in 2011.

I begin this study by reviewing the literature to expose some of the root causes of forced migration or what are called push factors. Beyond the scope of this article are all kinds of pull factors often related to the situation of forced migrants inside the host country, such as demand for labour, high wages, generous welfare benefits, good healthcare and education systems, strong economic growth, technology, a low cost of living, and social and cultural safety nets. Next, I demonstrate, using the example of Syria, how the neoliberal economic policies advocated by the IMF and the World Bank did not consider the dynamics of authoritarianism in the Arab Spring countries, and have altered the ‘social contract’, leading to civil wars and subsequently triggering a wave of forced migration from these states, particularly Syria. I go on to show how Syria's adoption of a wide range of liberal economic reforms aimed at transitioning to a more open market economy, as advised by the IMF and the World Bank, ultimately led to instability in three main ways. First, the reforms had a disproportionately negative impact on the agricultural sector, which was the primary source of employment for the majority of Syrians. Second, they resulted in the cutting of state subsidies and social services. Third, they marginalised young people, particularly those with higher levels of education, both economically and politically.

The three main questions addressed here are:

- How did the neoliberal economic policies, implemented with the support of the IMF and the World Bank, contribute to the destabilising of the Syrian state?
- How did these policies contribute to increased economic inequality, unemployment, and poverty, leading the state to decrease its provision of social welfare?
- How did these economic changes contribute to Syria's transition from reform to civil war and subsequently to forced migration?

## 2 THE ROOT CAUSES OF FORCED MIGRATION

In this section, I outline some of the root causes that push people away from their home, as indicated in previous studies.

### 2.1 Conflict, violence, and political instability

According to Howard (2004), refugees flee their country because of violent conflict and high levels of violence. Therefore, reducing the occurrence of violent conflict and the intensity of violent acts would help to alleviate the fears of many citizens and encourage them to stay rather than become refugees.

Lindley (2009) focused on the causes and processes of migration in Somali regions of the Horn of Africa, confirming that violent mass conflict disrupts the lives of millions of people. Migration often occurs due to violent conflict, with people moving within the affected area or country, and across borders to neighbouring states or further afield.

Moore and Shellman (2004) used a global sample of countries over the course of more than 40 years to study the impact of fear of persecution on forced migration. These authors contend that people monitor the violent behaviour of both the government and dissidents and assess the threat that it poses to life and liberty. They conclude that the greater the threat presented by the actions of the government and dissidents, the larger the number of forced migrants a country will produce.

Twomey (2014) studied displacement and dispossession owing to land grabs in Mozambique and found that they have created a new avenue via which people are being displaced and dispossessed of their land. He defines land grabbing as the transfer of land use rights or control over land, traditionally utilised by communities, to foreign investors for commercial purposes (frequently within the agricultural sector). It is often facilitated by partnerships with powerful domestic actors and justified by the investment stimulated.

As for current irregular migration to Europe, political instability in countries of origin is thought to be contributing to this inflow of people, including the conflict in Syria and crises in Libya and Tunisia (Cummings et al., 2015). According to Bradley (2017), though, the impact of violence on displacement may be direct or indirect: people may flee violence because it directly threatens their safety, their means of subsistence, or their home, or because it indirectly affects their life through its bearing on economic opportunities, social networks, and political institutions.

Conflict is a major driver of displacement, with violence shown to have direct and significant effects. Yet, two equally violent conflicts do not always generate the same level of displacement, and even in highly violent environments, not everyone flees; displacement is a decision, and some people choose to stay. Levels of conflict‐induced displacement have followed a general upward trend, with most of the increases due to higher numbers of internally displaced persons (IDPs); in contrast, refugee numbers have remained relatively stable. Most of those displaced by conflict do not cross an international border—they stay within their country of origin as IDPs.

### 2.2 Genocide and ethnic and civil conflicts

Many researchers state that genocide and ethnic and civil conflicts cause a refugee exodus. For instance, Fein (1993) claims that most refugee migrations were caused by some type of genocide against a particular group, such as the Dinka in Sudan, the Baha'is in Iran, or the Kurds in Iraq and Turkey. Since the end of the Second World War in 1945, some 50 episodes of mass killing have led to between 12 and 25 million civilian casualties, and by 2008, induced the displacement of 42 million people (Esteban, Morelli, and Rohner, 2015). For example, the mass killing in Sudan's Darfur region that started in 2003 caused between 70,000 and 400,000 fatalities, with an estimated 1.8 million people displaced (Esteban, Morelli, and Rohner, 2015).

Jenkins and Schmeidl (1995) demonstrated that revolutionary regimes are prone to using terroristic methods, including genocide, to rid themselves of political opponents. These shade into other types of ethnic warfare but are best represented in the contemporary period by the ethnic purification campaigns of Iraq against the Kurds, the Iranian persecution of the Baha'is, the Khmer Rouge's genocide against Cambodians, and Filipino attacks on Muslim Moros.

Ethnic and civil conflict can be promoted by a power struggle between the government and an insurgent group or between two equally large groups contending for power in a weak and unstable political environment. It frequently emerges when there are high levels of inequality and political exclusion. Civil conflict might be an alternative channel for people to express their grievances in societies that lack institutional means for broad political participation (Schmeidl, 1997; Kaufmann, 1998). Moreover, Newland (1993) found that ethnic diversity does not automatically produce conflict, and ethnic conflict does not automatically produce violence. Ethnic violence, however, very often produces refugees.

Genocide happens not only inside the origin countries of forced migrants; refugee camps also sometimes become a site of genocide against refugees. For instance, Palestinian refugee camps in Lebanon were destroyed in that country's civil war, such as Tel al‐Zaatar in 1976 (Aql, 1995; Salehyan and Gleditsch, 2006; Suleiman, 2006). Ruuska (2022) conducted a study in camps to which Rwandan refugees escaped after the genocide of 1994, with the aim of broadening understanding of genocide: from mass murder and direct physical violence to a process that entails multiple forms of violence, such as structural and psychological violence. She found that although refugee camps are supposed to be safe havens, genocide continues there.

### 2.3 Human rights violations

According to Lülf (2019), the root causes of refugee displacement are inextricably linked to conflict, persecution, and the denial of human rights. Buzurukov and Lee (2016) reported that an important driver of refugee migration is restrictions on and violations of civil liberties, which lead to human rights violations.

Davenport, Moore, and Poe (2003) conducted statistical analyses using fixed‐effects least squares on a pooled cross‐sectional time‐series dataset, consisting of data from 129 countries for the years 1964–89. They found that threats to personal integrity are of primary importance in a person's decision to abandon their home. Determinations of state threats to personal integrity, dissident threats to personal integrity, and joint state–dissident threats each have statistically significant and substantively important effects on forced migration.

According to Apodaca (1998), human rights abuses are a precursor to refugee flight, providing the immediate impetus. Gross human rights violations, in the form of torture, extrajudicial killings, disappearances, rape, and unlimited detention, create a general climate of fear that sparks abrupt departures. Refugee flights from Bosnia‐Herzegovina, Guatemala, the Horn of Africa, Iraq, and Myanmar have been directly attributed to human rights abuses.

### 2.4 Natural disasters

Natural disasters are a fundamental reason for forced migration, according to many studies (Nanda et al., 1984; Cohen and Werker, 2008). They are a reminder that threats to one's integrity can also come from exposure to natural hazards such as earthquakes and floods. The same is true of famines, which are often a consequence of the combination of adverse natural conditions and violent political conflict (Neumayer, 2005).

Climate change is increasingly seen as one of the major crises confronting the international community in the twenty‐first century and will soon become one of the leading causes of forced migration (Aminzadeh, 2007; Haris, 2020). For instance, by 2050, Bangladesh could lose 18 per cent of its land because of the rising sea level, resulting in 30 million displaced people or climate refugees. The displacement of people to new and existing settlements will put additional pressure on infrastructure and other services (Choudhury and Mowla, 2011).

Abel et al. (2019) examined the causal link between climate, conflict, and forced migration. Their results indicate that climatic conditions, affecting the severity of drought and the likelihood of armed conflict, played a significant role in asylum‐seeking in 2011–15. The effect of the climate on the occurrence of conflict was particularly relevant to countries in West Asia in 2010–12, when many of them were undergoing political transformation. The findings of Abel et al. (2019) suggest that the impact of the climate on conflict and asylum‐seeking flows is limited to specific periods and contexts.

Missirian and Schlenker (2017) examined how, in the recent past (2000–14), weather variations in 103 source countries translated into asylum applications in the EU. They found that temperatures that deviated from the moderate optimum (20°C) increased asylum applications, which implies an accelerated rise under the condition of continued future warming. In line with this study, Burke, Hsiang, and Miguel (2015) discovered that deviations from moderate temperatures and precipitation patterns systematically augment the risk of conflict.

Gleick (2014) concluded that the availability of water and climatic conditions played a direct part in the deterioration of Syria's economic conditions and the civil war that began in March 2011. Additionally, Kelley et al. (2015) reported that before the Syrian uprising commenced, the greater Fertile Crescent in the Middle East and North Africa experienced the most severe drought on record. For Syria, a country marked by poor governance and unsustainable agricultural and environmental policies, the drought had a catalytic effect, contributing to political unrest.

### 2.5 Development projects

Among the multifaceted causes of forced migration are projects purportedly undertaken for the benefit of society. This form of displacement arises from the implementation of large‐scale infrastructure and development schemes, ranging from the construction of highways, irrigation systems, power plants, and industries to urban expansion initiatives including hospitals, schools, and airports. While such programmes are widely recognised as essential for national development, economic modernisation, and the enhancement of public welfare, they simultaneously impose substantial costs on specific segments of the population.

Development‐induced displacement refers specifically to the involuntary physical relocation of individuals or entire communities due to state‐driven or corporate‐led development agendas. These initiatives may centre around dam construction, mining operations, transportation infrastructure, and urban renewal schemes, among others. Unlike displacement caused by war, violence, or natural hazard‐related catastrophes, development‐induced displacement stems from planned interventions with the intention of fostering progress and growth. Nevertheless, the impact of such displacement can be deeply destabilising. Cernea (2000) emphasises that although these projects aim to improve living standards and generate employment, the involuntary displacement they cause often results in severe social and economic disruption for the communities affected. Oliver‐Smith (2009) and Gupta and Marsing (2018) have pointed out that the scope of relocation varies, ranging from intra‐local movement (from one neighbourhood or village to another) to cross‐border displacement in regions that are economically, socially, and culturally unfamiliar.

Development‐induced displacement may unfold gradually over extended periods, often giving the illusion of manageable change. In many cases, however, it occurs abruptly, forcing people to leave their home and land with little or no notice—a point underscored by Terminski (2012) in his analysis of the human security risks associated with development projects.

The global scale of this phenomenon is staggering. It is estimated that approximately 15 million people are displaced each year because of public and private sector infrastructure projects, mostly in rapidly industrialising nations such as China and India, where the pace of development is particularly intense. Between 1947 and 2004, for example, India alone is estimated to have displaced nearly 60 million people for development purposes (Aboda et al., 2019).

While absolute numbers in Africa may appear modest in comparison, the relative impact on national populations is frequently far more profound. Cases such as the Akosombo Dam in Ghana, the Kossou Dam in Côte d'Ivoire, and the Kariba Dam on the Zambia–Zimbabwe border illustrate how development projects can displace a disproportionately large share of the population. As Rew, Fisher, and Pandey (2006) argue, the consequences of involuntary resettlement in African contexts may be even more severe, placing immense strain on both individuals and the state apparatus.

One of the most pervasive outcomes of development‐induced displacement is impoverishment. In his foundational work, Cernea (2000) introduced the impoverishment risks and reconstruction (IRR) model, which outlines eight core risks commonly associated with such displacement: (i) landlessness; (ii) joblessness; (iii) homelessness; (iv) marginalisation; (v) increased morbidity; (vi) food insecurity; (vii) loss of access to common property resources; and (viii) community disarticulation. Taken together, these risks show that development‐induced displacement can deepen hardship instead of delivering the promised benefits of progress.

### 2.6 Economic problems

Economic problems are seen as an accelerator of refugee flight. The United Nations (UN) system argues that economic factors lead to the underlying political causes of flight, whereas Schmeidl (1997) suggests that political and economic factors can interact in shaping refugee migration.

According to Malkki and Toole (1992), since the early 1960s, most emergencies involving refugees and displaced persons have taken place in less‐developed countries where local resources have been insufficient for providing prompt and adequate assistance. Furthermore, Robinson, Zimmerman, and Checchi (2014) found that between late 2010 and early 2012, southern and central Somalia experienced severe food insecurity and malnutrition precipitated by a prolonged period of drought, resulting in the poorest harvests since the famine of 1992–93. As a result of this emergency, large numbers of people were forced to move internally or migrate to already overcrowded refugee camps in Dadaab (Kenya) and Dollo Ado (Ethiopia).

McAdam (2009) concluded that the notion of economic persecution is neither modern nor radical. Historical evidence suggests that from entry into force of the 1951 Refugee Convention, some socioeconomic disadvantages, such as deprivation or systematic economic discrimination, were understood to constitute persecution under its framework.

Mutsvara (2024) investigated the impact of the economic liberalisation programmes designed by the IMF and the World Bank on cross‐border displacement in Sub‐Saharan Africa. He claims that various human displacement factors interconnected with economic liberalisation have become a new form of persecution, causing cross‐border displacement on the continent. He states that neoliberal policies in the form of structural adjustment programmes have negatively affected health determinants and slanted economic growth, threatening life and liberty in the countries that adopted them. The impact of cutting subsidies was very bad, for instance: the shrinkage of government services and the reduction in social spending offloaded responsibilities on to women, who are forced to look for alternatives to take care of family members when their spouse is out of work. Moreover, in three southern African countries (Malawi, Zambia, and Zimbabwe), daily protein consumption (per capita) fell by 20–25 per cent during the period between 1970 and 1995. This led to serious malnutrition in children and contributed to a high infant mortality rate. In addition, the life expectancy rate declined to 52 years, before hitting a low of 37 years for Zimbabweans in 2006.

Mutsvara (2024) underscores that while these economic packages have helped to establish markets, they have also caused abject poverty and had a negative bearing on human development. They have failed to provide a stable socioeconomic environment and by not aptly fitting the African landscape, they have resulted in more dependence on the West. He concludes that economic liberalisation has subverted African economies and in turn caused cross‐border displacement.

In the remainder of this paper, I show how applying the neoliberal economic policies advocated by the IMF and the World Bank to a number of Arab Spring countries resulted in the forced migration wave from 2010, especially in the case of Syria. To my knowledge, no other study has tackled this issue in the context of the Middle East and North Africa.

## 3 HISTORICAL PHASES OF TRANSFORMATION IN SYRIA

Prior to 2011, Syria experienced three major systemic transformations in its modern history, which can be classified as follows: (i) the post‐independence era (1946–49); (ii) the military coups era (1949–2000); and (iii) the neoliberal turn under Bashar al‐Assad (2000–24).

### 3.1 The post‐independence era (1946–49)

Following independence from French mandate rule in April 1946, Syria embarked on a turbulent but civilian‐led political phase characterised by intense parliamentary activity and ideological pluralism. The post‐independence years were marked by frequent changes in government, weak party coalitions, and growing tensions between nationalist and socialist currents. Despite these challenges, the period witnessed efforts to consolidate democratic institutions and expand state functions; however, the civilian political elite struggled to maintain cohesion amidst rising socioeconomic pressures and external influences. These dynamics culminated in the country's first military coup in March 1949, ending this short‐lived experiment in parliamentary governance (Perthes, 1995; Seale, 1989; Hinnebusch, 2001).

### 3.2 The military coups era (1949–2000)

Syria experienced a prolonged period of political instability and authoritarian consolidation shaped by successive military coups between 1949 and 2000. The first coup, led by General Husni al‐Za'im in March 1949, signified the military's entrance into Syrian politics. Multiple coups followed over the next two decades, reflecting intense power struggles among nationalist, Ba′athist, and Nasserist factions. The 1963 coup brought the Ba′ath Party to power, leading to increasing militarisation of governance. Hafez al‐Assad's ‘Corrective Movement’ coup in 1970 solidified a long‐standing authoritarian regime characterised by centralised control, suppression of dissent, and the establishment of a powerful security apparatus. Under Hafez al‐Assad's rule (1971–2000), Syria became a one‐party state dominated by the Ba′ath Party and loyalist military elites (Heydemann, 1999; Hinnebusch, 2001).

### 3.3 The neoliberal turn under Bashar al‐Assad (2000–24)

On assuming power in 2000, Bashar al‐Assad initiated a series of market‐oriented reforms aimed at liberalising Syria's state‐controlled economy. This neoliberal turn included the opening of private banks, the reduction of subsidies, the promotion of foreign investment, and partial privatisation of state assets. While these reforms were intended to modernise the economy, they disproportionately benefited regime‐connected elites and exacerbated socioeconomic inequalities, particularly in rural areas. The dismantling of agricultural subsidies and the weakening of social safety nets contributed to rising poverty and unemployment. Combined with endemic corruption and authoritarian governance, these policies deepened public discontent, laying the groundwork for the 2011 uprising (Ismail, 2011; Haddad, 2012; Hinnebusch, 2012).

While the first two phases of Syria's modern history—the post‐independence and the military coups eras—played a crucial role in shaping the political and social fabric of Syrian society, including the establishment of a military dictatorship and the reinforcement of ethnic and sectarian divisions, this study focuses primarily on the third phase: the neoliberal turn under Bashar al‐Assad (2000–24). This period represents the culmination of long‐standing developments in Syrian governance and society.

The emphasis on this neoliberal transformation does not imply a disregard for the significance of earlier historical developments or other influential factors. On the contrary, the legacy of authoritarian rule, entrenched sectarian dynamics, and the evolving role of digital technologies—particularly the mobilising power of social media platforms such as Facebook—are considered to be very important in understanding the 2011 uprising and the resulting mass displacement of Syrians.

## 4 THE ‘SOCIAL CONTRACT’ IN POST‐INDEPENDENCE SYRIA—EXCHANGING POLITICAL FREEDOM FOR GOODS, CERTAIN SERVICES, AND JOB OPPORTUNITIES

Before the Second World War, state management was in the hands of colonial great powers and the local upper classes related to them, mainly from urban cities. After becoming an independent nation‐state in 1946, however, Syria, as well as Egypt and Iraq for that matter, witnessed massive changes at the beginning of the 1950s and 1960s. Notably, upper‐class civilian politicians, who were educated in European academic institutions, were replaced by young officers from the lower classes or rural areas of society (Fedai, 2017).

After the coup in 1963, military officers in Syria established an authoritarian state that privileged rural peasants, urban public workers, and minorities at the expense of the traditional land‐owning class and the Sunni bourgeoisie in the big cities (Mora and Wiktorowicz, 2003). These military officers, who were members of the Ba′ath Party, followed up the coup immediately with a social revolution aimed at improving the material conditions of rural communities, which had suffered under both French mandatory and independent rule (Nelson, 1998).

This new elite, whose worldview was shaped by their rural background, managed to instigate what was called a ‘revolution from above’ that broke the economic hold of the oligarchy, won the support of peasants via land reform, and created through nationalisation a public sector that employed major segments of the middle and working classes (Hinnebusch, 2012). Thus, the stability of the state was dependent on labourers and peasants, who constituted the backbone of the regime's legitimacy.

The Syrian economy up to now had been characterised by state dominance over the main productive sectors and major state interference in economic activity. The state dealt a devastating blow to large properties, through the confiscation of agricultural lands or by acquiring ownership of industrial and commercial companies, through nationalisation and seizures. In 1970, the public sector comprised 70 per cent of the economy and the private sector made up 30 per cent of it (Hinnebusch, 1995). Moreover, the state controlled the entire manufacturing base, and most foreign trade, and became a major player in relatively widespread internal trade, especially in petroleum derivatives and grains, flour, and cotton.

Health services were provided free of charge and public health centres expanded in rural areas. Education was free, including university. Furthermore, a relatively generous energy and food subsidies system was put in place. The agricultural sector received state support through guaranteed purchasing of ‘strategic’ crops, subsidies for fertiliser, energy, and machinery, and exemption from the payment of taxes (Azmeh, 2016).

Many people benefited from this social revaluation in Syria. For instance, investment in public health and education led to literacy and life expectancy increases that continued into the 1990s. Electrification in rural areas rose from 2 per cent in 1963 to 95 per cent in 1992, reflecting the regime's support base (Hinnebusch, 2012; Fedai, 2017).

The ‘social contract’ is an idea that dates back to the ancient Greeks, referring to the implicit agreement among members of a society that defines their relationship with each other and the state (Devarajan, 2015; Sibun, 2022). The dominant aspect of the social contract developed in Syria, after the military coup in 1963, was that the population resigned itself to a lack of political freedom in exchange for the provision of certain services, like state employment, access to public healthcare and education, and exemption from or low taxation (Hopfinger, 1996; UNDP, 2011).

These developmentalist policies, however, created hope only for a while. Controlling economic sources allowed the state to increase its involvement in society and regulate it in such a way as to strengthen its position. New housing, roads, public services, schools, hospitals, and public works increased the welfare level of the people, but this situation could not be sustained. Corruption and ineptitude became common in the rapidly expanding public sector—an unavoidable scenario in political systems based on a patron–client relationship. As economic improvement decreased, the demand for welfare used up existing resources and the state started to become indebted (Fedai, 2017).

It is important to note that this form of social contract—where political freedom is exchanged for the provision of goods, public services, and employment—proved relatively sustainable and, in some cases, successful in fostering development across certain post‐colonial Arab states. In oil‐rich countries, where the state had greater financial resources—such as the Gulf states and Libya—this model delivered measurable developmental outcomes and maintained stability for longer periods. In nations like Libya, for instance, it achieved significant developmental gains before being forcibly dismantled. In other contexts, though, including Egypt, Syria, and Tunisia, where economic resources were more limited, the model ultimately failed. Its collapse not only led to internal instability but also opened the door for external actors, including the IMF and the World Bank, to intervene and implement structural adjustment programmes. The implications of these interventions are discussed in more detail in the following section.

## 5 ECONOMIC CRISES AND APPLYING NEOLIBERAL ECONOMIC POLICIES IN SYRIA

From the early 1990s, the Syrian economy found itself in crisis owing to the pressure of internal and external factors. The most pertinent external reason was the collapse of the Soviet Union. For about 35 years, Syria followed economic policies modelled on the Soviet experience, but after the demise of the Eastern Bloc in 1991, the government started moving hesitatingly towards a market economy (Murden, 2005; Seifan, 2011).

The most prominent internal factor was inefficient central planning for the economy. Mora and Wiktorowicz (2003) show that major state involvement in the economy produced inefficient bureaucracies and public enterprises riddled with corruption, inadequacies, rent‐seeking, and nepotistic practices designed to bolster loyalty. Consequently, the public sector failed as an engine of capital accumulation because it was used to provide populist benefits such as jobs, food, and patronage to the regime's constituency. Alienated private capital fled the country or refrained from investment, except in tertiary sectors that yielded quick profits (Hinnebusch, 2012).

In response to the severe economic crisis from the early 1990s, the government implemented economic reforms designed to move the Syrian economy towards free‐market capitalism (Nelson, 1998). Fedai (2017) concluded that Arab nations, including Syria, understood that central economic planning was not an effective economic method. The state had no property to nationalise and oil prices decreased after a rapid increase. This situation put neoliberal policies on the agenda of many Arab countries.

The idea of economic reform is called *infitah* in Arabic (Murden, 2005). Literally meaning ‘opening’, this term is widely used to describe economic liberalisation throughout the Middle East (Nelson, 1998). The problem, though, is that *infitah* represented yet more distorted development. Reform was patchy and the benefits did not extend very far into society. The new markets were rigged for the advantage of an emerging class of private oligarchs and their bureaucratic allies within the state. The economics of crony capitalism were largely based on political and social criteria rather than market principles. Business ties were frequently cemented by personal, familial, and marital alliances, while the decisions of state regulators significantly determined the capacity of those who were able to make profits (Murden, 2005). Furthermore, *infitah* reforms raised the spectre of social inequality. The new public–private elites may or may not have continued to pay lip service to old populist ideologies, but in practice, they tended to aspire to Western lifestyles that were far removed from the reality of ordinary people.

Bashar al‐Assad came to power in Syria (in 2000) at a particularly dangerous time for the Arab states. The West was simultaneously exporting neoliberalism as the only legitimate economic course, which in Arab countries meant favouring investors and deconstructing the populist social contract by which the republics had legitimised themselves, and promoting democratic discourse that tended to delegitimise authoritarian regimes, particularly once they departed from their initial social contract. Bashar al‐Assad's project, on his accession to the presidency, was to open up the economy to the world market and adapt the country to enter the age of globalisation (Hinnebusch, 2012).

These economic policies created more opportunities for private investors. The passing of new laws allowed private investment in sectors such as telecommunications, banking, insurance, real‐estate development, and education. This was followed by the launch of the Damascus Securities Exchange in 2009 (Azmeh, 2016).

Owing to the reform policies, the private sector was able to increase its share of the Syrian economy. In 2007, the private sector's proportion reached around 60.5 per cent of gross domestic product (GDP) (Haddad, 2011).

The World Bank stated that its interest in privatisation stems from its fundamental goal to help borrowers achieve efficient growth with equity while reducing poverty and protecting the environment—that is, an important means to these ends (Shirley, 1991). Yet, like most of the structural adjustment programmes at the time, the main objectives of the prescribed reforms were to decrease foreign debt and inflation by cutting state subsidies, privatising public companies, liberalising markets and prices, freezing wages, and commercialising agricultural lands (Öztaş, 2020).

Syria, although a relative latecomer to economic reform, followed this same, prescriptive neoliberal economic reform methodology under Bashar al‐Assad, with the cutting of government spending a primary focus. These decreases in expenditure, especially in the realm of social services, would prove dire in Syria because of the additional strain placed on Bashar al‐Assad's regime and the Syrian people at large by a subsequent drought. This event put Syria in extremis; a central response was needed, but the Syrian state was both unwilling and unable to do so because of the economic liberalisation policies enacted (Guffey, 2017).

Under its *Articles of Agreement* with Syria, the IMF is to hold bilateral discussions and produce an annual overview of the country's economic health and vitality. The last *Article IV Consultation* completed for Syria was released in spring 2010 (International Monetary Fund, 2010). It is reported that the Syrian authorities have been implementing gradual but wide‐ranging reforms, motivated by the challenges posed by the decline in oil production and the strategy initiated in the early 2000s to transition to a social market economy. The exchange rate has been effectively unified and restrictions on access to foreign exchange appear to have been mostly eliminated for current transactions. Private banks are now leading growth in the financial sector and the Damascus Securities Exchange, which opened in 2009, has recently become operational. Taxes have been streamlined and the trade regime has been significantly liberalised. Moreover, an emphasis on further subsidy reform is prevalent throughout the report, specifically agricultural subsidy cuts (Guffey, 2017).

In addition, negotiations on an EU–Syria Association Agreement, widening economic and commercial ties, relations with international organisations, and even diplomatic links are also putting constant pressure on policymakers to move faster towards the realisation of a market economy. A conviction has evolved that a market economy and an expanding role for the private sector will ultimately contribute to improving Syria's relations with Western European countries (Seifan, 2011).

## 6 SOME SIGNIFICANT CONSEQUENCES OF APPLYING NEOLIBERAL ECONOMIC POLICIES IN SYRIA

### 6.1 Marginalisation of the agricultural sector and rural populations

Syria is largely dependent on agriculture, with a total cultivated area of about 4.9 million hectares. Agriculture contributes more than 26 per cent of GDP and employs 19 per cent of the total population (Bazza, Kay, and Knutson, 2018). Yet, in the years since 2000, the agricultural sector in Syria has been the gravest casualty of both economic reform and drought (Haddad, 2011).

In an increasing number of research projects and policy assessments, many experts are beginning to make the case that underlying environmental factors can be blamed for sparking the civil war in Syria (Voski, 2016; Bazza, Kay, and Knutson, 2018; Read, 2019). According to Kelley et al. (2015), as noted earlier, before the Syrian uprising began in 2011, the greater Fertile Crescent experienced the most severe drought on record. For Syria, a country marked by poor governance and unsustainable agricultural and environmental policies, the drought had a catalytic effect, contributing to political unrest. Some researchers, though, have drawn a different conclusion, namely that: there is no clear and reliable evidence that anthropogenic climate change was a factor in Syria's pre‐civil war drought; this drought did not cause anywhere near the scale of migration that is often alleged; and there is no solid evidence that drought migration pressures in Syria contributed to the onset of civil war (Hsiang and Burke, 2014; Selby et al., 2017).

The economic policy during the transition to a social market economy included the dismantling of the structure that had long supported the agricultural sector in Syria and the rural populations that worked in it. As part of an overall transition by the state away from the agricultural sector and towards other non‐agricultural sectors that it was thought would generate more capital, the subsidies for agricultural inputs (such as fertilisers, fuel, and seeds) were removed. These adjustments were combined with a reduction in state support, in the form of social welfare to rural communities, placing considerable strain on those Syrians who traditionally worked in the agricultural sector (Guffey, 2017).

**FIGURE 1:**
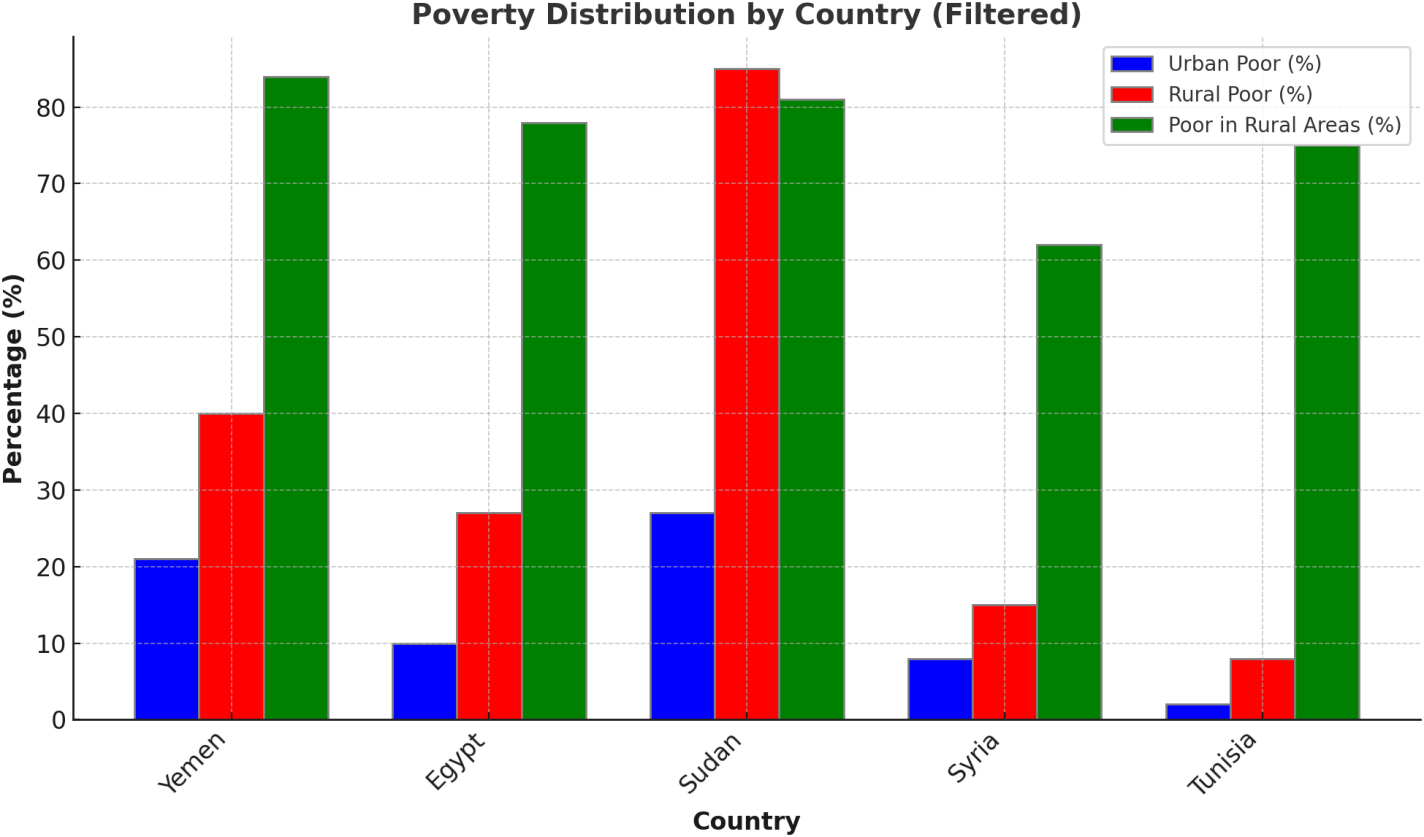
Poverty distribution by country. **Source:** author, using data from Lampietti et al. (2009).

Syria, together with several other Arab states, have vehemently marginalised rural areas in general developmental projects. Infrastructure and services have been neglected therein, increasing poverty rates (see Figure 1). For instance, the rural populations in 6 of the 22 Arab countries—Egypt, Jordan, Morocco, Syria, Tunisia, and Yemen—constitute 53 per cent of the total population, yet their share of poverty is 74 per cent (UNDP, 2009). In 2005, for instance, approximately 20.3 per cent of the Arab population was living below the international poverty line of USD 2 per day. This estimate is based on seven Arab middle‐ and low‐income groups, whose population represents about 63 per cent of the total population of Arab countries not in conflict. Using the international poverty line indicates that, in 2005, about 34.6 million Arabs were living in extreme poverty. The situation is especially acute in low‐income Arab countries, where some 36.2 per cent of the population is living in extreme poverty. Expectably, income poverty, and the insecurity associated with it, are more widespread among rural populations (UNDP, 2009). Consequently, the Arab region in general has the largest food deficit in the world and depends greatly on food imports, widening the debt base and economic dependence. As food prices continue to spike, food subsidies become an unsustainable fiscal policy tool for most Arab countries (Dagher, 2015).

In Syria, employment in the agricultural sector decreased from 28.3 per cent in 1995 to 14.8 per cent in 2010 (Sika, 2013). Moreover, among the governates that depend on agriculture, the northeast region (Al‐Hasakah, Al‐Raqqah, Aleppo, Deir Ez‐Zor, and Idlib) was the poorest area in the country right before the uprising, according to a report issued by the Ministry of Agriculture and Agrarian Reform in 2010 (Ferkel, 2010). Hence, the economic reform policies that were implemented in Syria and some non‐oil Arab countries changed the deep structure of the economy, from one based on agriculture and manufacturing industry to one centred on services. The share of services was higher than 50 per cent in non‐oil‐producing countries, such as Egypt and Syria.

Fedai (2017) found that food prices in the Arab World reached their highest level since 1990 in December 2010, and that the 2010 rise in oil prices led to the removal of most fuel subsidies. Together with the 2008 financial crisis, these factors contributed to the uprisings in 2011.

### 6.2 Cutting state subsidies

As shown above, during the post‐independence era, important components of the Syrian political regime's legitimacy were subsidies and social expenditures. Yet, the IMF and the World Bank insisted that states deal with them to decrease foreign debt and inflation. In the neoliberal reform period, therefore, social expenditure started to decrease, impacting deeply on the social contract between Syrians and their government.

This happened not only in Syria but also in all Arab Spring countries. For instance, during the 2000s, the rate of public expenditure as a proportion of GDP declined severely in Egypt and Tunisia. The main reason for this was the decrease in public sector expenses. In Egypt, while subsidies made up 14 per cent of public expenditure between 1980 and 1981, this proportion fell to 5.6 per cent between 1996 and 1997. To realise this reduction, the Egyptian government introduced different strategies, such as lowering the number of subsidised goods to four (taking meat, fish, and rice off the list), cutting the number of people receiving aid, allowing the prices of sugar and bread to increase, and lessening the quality and quantity of supported products. In Tunisia, reductions in the number of subsidised products and other changes left an important section of urban and rural poor people beyond the scope of aid plans (Fedai, 2017).

**FIGURE 2:**
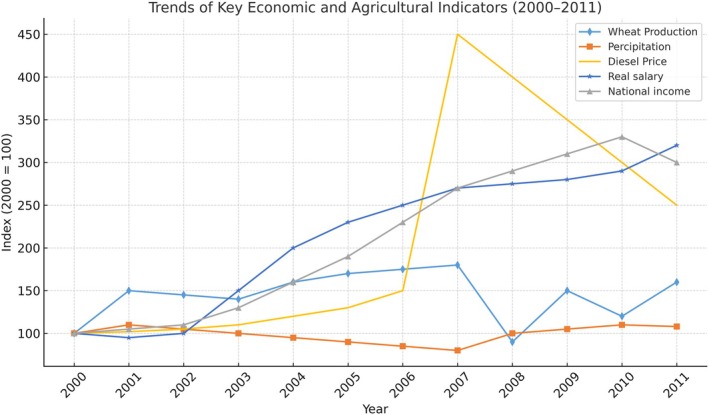
Changes in economic indicators and the precipitation level (per cent, based on the year 2000). **Source:** author, using data from Lampietti et al. (2009).

Almohamed and Birner (2019) studied the consequences of the diesel subsidy cut in Syria and determined that the new diesel price policy (May 2008) had radically affected the whole national economy (see Figure 2). The sudden increase in the diesel price (of about 257 per cent) led to the production costs of crops increasing by more than 50 per cent, which caused a huge decline in gross margins and forced farmers in the irrigated region to leave their villages and migrate from the northeast region to the cities in search of new jobs. The diesel subsidy cut also resulted in the shutting down of hundreds of private factories, a dramatic fall in domestic and foreign trade, and the inflation rate reaching 15 per cent.

Unquestionably, these steps were detrimental to the prevailing social contract in the region, especially in middle‐ and lower‐income countries like Egypt, Syria, Tunisia, and Yemen, where governments provided for the socioeconomic well‐being of citizens in return for authoritarian regimes remaining in power. Government subsidies and public enterprises were essential for sustaining the economic well‐being of the lower and middle classes. Moreover, government enterprises ensured the employment of middle‐class technocrats. Hence, with the structural adjustment policies of the international financial institutions, aimed at reducing the public sector, a large number of government supporters became unemployed and blamed the central authorities for their plight (Hopfinger, 1996).

Thus, economic reform efforts threaten the social contract that sustains regime coalitions. In non‐democratic regimes generally, market distortions are frequently used to promote political support among key social and economic actors. Patronage is channelled through targeted subsidies, investment opportunities, public employment, currency manipulation, and other interventionist policies. Economic liberalisation weakens the politicisation of the economy by eliminating some of these distortions through privatisation and structural adjustment (Mora and Wiktorowicz, 2003).

The economic reforms in Syria encouraged the establishment of private schools, universities, and medical facilities for the new rich, paralleled by a precipitous running down of public services for ordinary citizens. This was emblematic of the changing social base of the regime (Hinnebusch, 2012).

Despite such a radical decrease in social spending and its obvious impacts on the welfare and food security of the population, the government failed to replace subsidies with any alternative viable social policy, introducing a small, poverty‐targeted scheme instead. It is likely that the abandonment of those people on middle incomes, who had previously been protected by the government and represented about 80 per cent of the population, triggered the popular discontent that eventually led to civil war (Sibun, 2022). Guffey (2017) concluded that as Syria transitioned from a state‐run economy to a participant in the global market, the government rolled back on social programmes that were previously the purview of the state. It was this lack of provision of social services that greatly influenced the people's decision to rise up.

### 6.3 Marginalisation of the youth population

**FIGURE 3:**
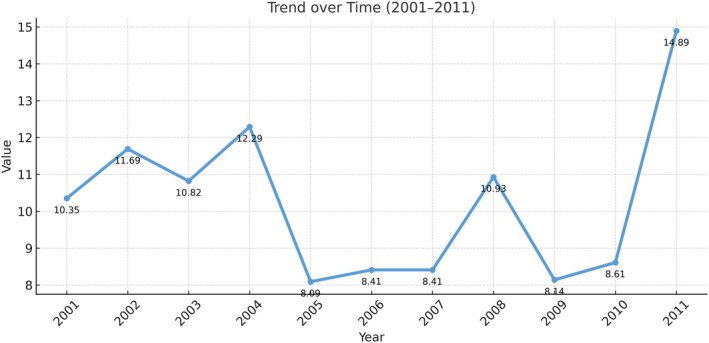
Unemployment rates in Syria. **Source:** author, using data from the Syrian CBS (2009).

Young people (aged less than 30) constitute more than one‐half (55 per cent) of the population in Arab countries. Yet, they are highly marginalised, especially in the employment market (see Figure 3). After the adoption of neoliberal economic measures, lower‐ and middle‐income Arab countries faced a grave problem: the increasing inability of the state to co‐opt the educated youth into the public sector. For example, in 2006, approximately 40 per cent of Egyptians were between the ages of 10 and 29, and about 25.6 per cent of Egypt's youth population that holds a higher education degree was unemployed in 2009. This happened due to the stagnation of development and economic productivity (Hopfinger, 1996; Johnson, 2014).

According to Sibun (2022), there were not enough jobs in the private sector to absorb the surplus demand in the labour market, as many governments had assumed there would be when they shifted to a market‐led strategy and adopted radical economic restructuring plans. At the same time, the youth population in many countries was growing. A ‘youth bulge’ emerged: young adults aged 24 years or less as a proportion of the population of Arab countries reached 50 per cent on average in 2015.

A common feature of the protests in Syria was that the people involved were in the 18–24 age group, or the extended 18–35 age group, which are the most vulnerable and take the initiative more than anyone else. They were facing the highest unemployment rates and included new youth groups that were interacting with ‘the winds of revolution’ sweeping across other Arab countries (Barout, 2011).

Unemployed youths with high levels of human capital but without the ‘connections’ with the authorities to attain employment or a decent salary in the private or public sector therefore became frustrated with the system. The sense of betrayal and victimhood was particularly evident among the young. A youth revolt against the state, private capitalists, and foreign interests was in the making (Hopfinger, 1996; Murden, 2005). Moreover, youths were also marginalised in the political sphere, where the dominant political parties, especially in Egypt, Syria, and Tunisia, were composed of business tycoons and individuals who had direct contact with the rulers.

In other words, the youth enter society and are immediately hit hard: high rates of unemployment; few openings for political participation; little hope for change; a harshly repressive state; and a repressive sociocultural superstructure. The newly educated poor were a particular case in point, with their social mobility blocked by fewer public sector jobs and by a private sector monopolised by the new class of public–private oligarchs.

The impact of the marginalisation of youth becomes especially clear when one looks at the demographic makeup of those who engaged in the Arab Spring protests—it was mainly those in the ‘youth bulge’ age group that took to the streets. Johnson (2014) pointed out that not only is the population of the region growing three times faster than in the rest of the world, but it is also becoming comparatively more educated than in past years. A greater percentage of the Arab population is earning a university degree. A population within which a significant portion of the people have received a degree in higher education is more likely to express discontent when economic or political conditions weaken.

## 7 NEOLIBERAL ECONOMIC POLICIES, THE UPRISING, AND THE SYRIAN REFUGEE WAVE

Neoliberal economic policies brought some economic growth to the Arab countries. For instance, according to World Bank estimates, the Middle East and North Africa region achieved an average annual economic growth of 6.2 per cent between 2003 and 2006. Yet, this GDP was highly volatile: Arab non‐oil‐producing countries have increasingly based their growth on import‐oriented and service‐based economies. The share of services was greater than 50 per cent. This type of economy flourished at the expense of agriculture, manufacturing, and industrial production (Hopfinger, 1996; Azmeh, 2016).

Moreover, although economic liberalisation was important for economic development, these countries established ‘crony capitalist’ systems, which were unable and unwilling to include the highly eroding middle class and the high number of socially and economically excluded youth. In Syria, the flow of international finance only benefited a small, elite circle strongly linked to the regime, exacerbating sectarian tensions in the country (Guffey, 2017).

The uprising events in the Arab Spring countries reflect the major influence of the economic reform policies. Notably, the 2011 protests started in Sidi Bouzid, Tunisia, a rural area. In Syria, although the government's economic policies led to faster rates of growth in the 2000s, they failed to address the key structural challenges facing the economy. The people's uprising started in Daraa, an agricultural area deemed to be a bastion of the regime in the post‐independence era. Through the process of urban boom and decline, the regime's old rural socioeconomic base was being eroded while a small new support base in urban and middle‐income areas was being created.

The influence of the agricultural sector was very important in the Arab Spring uprisings. An increase in the population and unemployment, political, economic, and ecological polarisation, and continuous exploitation pushed farmers in rural areas into extreme poverty. In Syria, 62 per cent of the poor population live in rural areas and 77 per cent of these people have no land. After applying economic reforms, the government did very little to meet the demands of those residing in rural areas. In contrast, the big cities that benefited from the economics of services stayed neutral or silent. For instance, Aleppo and Damascus remained relatively silent because the upper class of the business world was a strict supporter of the regime owing to interwoven interests (Fedai, 2017).

Hinnebusch (2016) observes that the opposing sides in the Syrian uprising reflected the regime's reconfiguration of its social base. The protests began in the deprived rural towns and suburbs and then spread to medium‐sized cities, such as Hauran, where small manufacturers were victims of trade liberalisation. The main cities of Aleppo and Damascus, where investment and consumption were concentrated, remained largely quiescent months into the uprising. Hence, Pace and Cavatorta (2012) found that when one analyses the Arab awakening, it is clear that ordinary citizens rose up against rigged neoliberal reforms imposed by Western organisations like the IMF and the World Bank, which led to an even more unequal distribution of wealth in Arab countries and impoverished the masses over two decades.

As has been observed, the protest movement had a clear class‐based nature; because it occurred predominantly in poorer rural and rural–urban areas. Azmeh (2016, p. 501) has described it as a ‘rural and urban revaluation’. The most violent episodes of the Syrian civil war have taken place in peripheral cities, such as Daraa and Douma, which suffer primarily because of multidimensional marginalisation, oppression by local authorities, repression by an arbitrary central government, and limited benefits from economic growth. These cities have low human development indicators, high rates of unemployment and poverty, and high age dependency (Barout, 2011; Dahi and Munif, 2012).

## 8 CONCLUSION

Researchers have identified a wide range of root causes of forced migration, including conflict, violence, political instability, genocide, ethnic and civil strife, human rights violations, disasters triggered by natural hazards, development projects, and economic hardship. This article has placed particular emphasis on the oft‐overlooked role of neoliberal economic policies—promoted by institutions such as the IMF and the World Bank—in precipitating civil unrest in Syria and contributing to the broader displacement of populations across the Middle East and North Africa following the Arab Spring of 2010.

Despite the extensive body of literature on forced migration, there remains a significant gap concerning the impact of structural adjustment and economic liberalisation programmes on state destabilisation, the outbreak of civil conflict, and subsequent population displacement. This study contributes to efforts to fill that lacuna by demonstrating how neoliberal reforms can create conditions that foster unrest, particularly in non‐democratic states with fragile institutions and limited engagement of civil society. It is hoped that this exploratory analysis will prompt further work on the intersection between economic restructuring and forced migration in other regions experiencing civil wars, social fragmentation, and refugee crises.

The findings suggest that neoliberal economic policies—often mandated by international financial institutions—played a pivotal role in igniting popular uprisings in Arab Spring countries. These reforms undermined local economies by disarticulating traditional production systems, dismantling public sector support, and reducing state expenditure on essential social services. In doing so, they exacerbated poverty, inequality, and unemployment, while marginalising vast segments of the population—including rural communities, the urban poor, and unemployed youths—which became increasingly alienated from state institutions and deprived of viable livelihood opportunities.

This analysis resonates with the work of Cypher and Delgado Wise (2011), Delgado Wise (2013), and Delgado Wise, Márquez Covarrubias, and Puentes (2013), who have drawn similar conclusions regarding the effects of neoliberal restructuring in Latin America, particularly in Mexico. They argue that these reforms have led to the dismantling and disarticulation of national productive structures—not only in agriculture but also, and crucially, in national industry. Through the privatisation of public enterprises and the retreat of the state from social and economic functions, a massive surplus population has emerged—displaced from productive activity and absorbed into informal economies or pushed into migration. In this context, multinational corporations play a central role as drivers and beneficiaries of this restructuring, highlighting the global power asymmetries embedded in the process. As Delgado Wise (2013) notes, these dynamics have produced a growing mass of ‘redundant’ people, no longer incorporated in national development strategies, and compelled to seek livelihoods elsewhere, often via migration.

These insights strengthen the argument for expanding the conceptual boundaries of forced migration. As Cypher and Delgado Wise (2011) observed, increasing migration flows from the Global South—such as the caravans crossing Mexican territory—testify to the fact that many people are fleeing not conventional war or persecution, but structural economic violence. These migrants, uprooted by neoliberal restructuring, fall outside of the conventional legal categories of refugees and asylum‐seekers, yet their displacement is no less compelled. A broader conceptual and policy framework is thus urgently needed—one that accounts for the role of global economic forces and neoliberal governance in generating new forms of forced migration rooted in systemic inequality and exclusion.

To mitigate future civil conflicts and refugee crises, it is imperative that institutions like the IMF and the World Bank reconsider the political and social contexts of the countries in which they implement economic reform programmes. Policies that focus solely on fiscal austerity and liberalisation, without addressing the need for inclusive growth and social protection, risk exacerbating state fragility. In non‐democratic regimes, the removal of subsidies and public goods may further undermine state legitimacy, especially where political freedoms are limited and civic dissent is suppressed. The adverse consequences of economic marginalisation are only intensified when coupled with political exclusion and authoritarian rule.

In sum, there is a pressing need to reassess critically the global economic order that contributes to forced migration. A deeper understanding of how neoliberal reforms shape migration flows is essential for developing more just and effective policy responses—both at the national and international level.

Emphasising the role of neoliberal economic policies in destabilising Arab Spring countries—and subsequently driving waves of forced migration—should not be taken as an attempt, though, to discount other significant contributing factors. The uprisings were also shaped by the enduring crisis of authoritarian governance, the mobilising power of social media platforms such as Facebook, rapid and poorly managed urbanisation, and deep‐rooted ethnic and sectarian divisions. This study, therefore, highlights one critical dimension within a broader constellation of interrelated causes that have evolved over decades. No single factor, however significant, can fully account for the complexity of the events that unfolded. Consequently, there is a clear need for further interdisciplinary research to deepen our understanding of the multifaceted drivers of the Arab Spring uprisings and the resulting patterns of forced migration.

## CONFLICT OF INTEREST STATEMENT

The author confirms that there are no conflicts of interest.

## Data Availability

The data that support the findings of this study are available on request from the corresponding author. The data are not publicly available due to privacy or ethical restrictions.
